# Supplementary material for: Gut microbiota-bile acid crosstalk contributes to the rebound weight gain after calorie restriction in mice
Source: Nat Commun. 2022 Apr 19;13:2060. doi: 10.1038/s41467-022-29589-7 (PMC9018700; doi:10.1038/s41467-022-29589-7)
Supplement: Supplementary file 1 — Supplementary Information [file 41467_2022_29589_MOESM1_ESM.pdf]

Gut microbiota-bile acid crosstalk contributes to the rebound weight gain after calorie restriction in mice

Supplementary information

Supplementary Table 1. The sequences of the strain.

Bacteria sequencing result
GCGACCGGCGCACGGGTGAGTAACGCGTATGCAACTTGCCTATCAGAGGGGGATAACCCGGCGAA<br>AGTCGGACTAATACCGCATGAAGCAGGGATCCCGCATGGGAATATTTGCTAAAGATTCATCGCTGAT<br>AGATAGGCATGCGTTCCATTAGGCAGTTGGCGGGGTAACGGCCCACCAAACCGACGATGGATAGGG<br>GTTCTGAGAGGAAGGTCCCCCACATTGGTACTGAGACACGGACCAAACTCCTACGGGAGGCAGCA<br>GTGAGGAATATTGGTCAATGGCCGAGAGGCTGAACCAGCCAAGTCGCGTGAGGGATGAAGGTTCT<br>ATGGATCGTAAACCTCTTTTATAAGGGAATAAAGTGCGGGACGTGTCCCGTTTTGTATGTACCTTATG<br>AATAAGGATCGGCTAACTCCGTGCCAGCAGCCGCGGTAATACGGAGGATCCGAGCGTTATCCGGAT<br>TTATTGGGTTTAAAGGGTGCGTAGGCGGCCTTTTAAGTCAGCGGTGAAAGTCTGTGGCTCAACCAT<br>AGAATTGCCGTTGAAACTGGGGGGCTTGAGTATGTTTGAGGCAGGCGGAATGCGTGGTGTAGCGGT<br>GAAATGCATAGATATCACGCAGAACCCCGATTGCGAAGGCAGCCTGCCAAGCCATTACTGACGCTG<br>ATGCACGAAAGCGTGGGGATCAAACAGGATTAGATACCCTGGTAGTCCACGCAGTAAACGATGATC<br>ACTAGCTGTTTGCGATACACTGTAAGCGGCACAGCGAAAGCGTTAAGTGATCCACCTGGGGAGTAC<br>GCCGGCAACGGTGAAACTCAAAGGAATTGACGGGGGCCCGCACAAGCGGAGGAACATGTGGTTT<br>AATTCGATGATACGCGAGGAACCTTACCCGGGTTTGAACGCATTCGGACCGAGGTGGAAACACCTT<br>TTCTAGCAATAGCCGTTTGCGAGGTGCTGCATGGTTGTCGTCAGCTCGTGCCGTGAGGTGTCGGCTT<br>AAGTGCCATAACGAGCGCAACCCTTGCCACTAGTTACTAACAGGTTAGGCTGAGGACTCTGGTGGG<br>ACTGCCAGCGTAAGCTGCGAGGAAGGCGGGGATGACGTCAAATCAGCACGGCCCTTACATCCGGG<br>GCGACACACGTGTTACAATGGCGTGGACAAAGGGAGGCCACCTGGCGACAGGGAGCGAATCCCC<br>AAACCACGTCTCAGTTCGGATCGGAGTCTGCAACCCGACTCCGTGAAGCTGGATTCGCTAGTAATC<br>GCGCATCAGCCATGGCGCGGTGAATACGTTCCCGGGCCTTGTACACACCGCCCGTCAAGCCATGGG<br>AGCCGGGGGTACCTGAAGTCCGTAACCGAAAGGATCGGCCTAGGGTAAAACTGGTGACTGGGGCT<br>AA

Supplementary Table 2. qPCR primers sequences.

Gene name	Primer sequence
16s	F: 5'- TCCTACGGGAGGCAGCAGT-3'<br>R: 5'- GGACTACCAGGGTATCTAATCCTGTT-3'
Parabacteroides distasonis	F: 5'-TGCCTATCAGAGGGGGATAAC-3'<br>R: 5'- GCAAATATTCCCATGCGGGAT-3'
Gapdh	F: 5'-TGCACCACCAACTGCTTAG-3'<br>R: 5'-GATGCAGGGATGATGTTC-3'
Gcg	F: 5'-GCCCAAGATTTTGTGCAGTGG-3'<br>R: 5'-GTCCCTTCAGCATGCCTCTC-3'
Tgr5	F: 5'-GCCCAAAGGTGTCTACGAGT-3'<br>R: 5'-TCAAGTCCAGGTCAATGCTG-3'
Cyp27a1	F: 5'-TCCCAGTGTCTTTCCTGAGC-3'<br>R: 5'-CACAGAGCCGAATGGATGTA-3'
Cyp7a1	F: 5'-CTGGGCTGTGCTCTGAAGT-3'<br>R: 5'-GGGAGTTTGTGATGAAGTGGA-3'
Cyp8b1	F: 5'-ACAGCGTGATGGAGGAGAGT-3'<br>R: 5'-AGGGGAAGAGAGCCACCTTA-3'
Cyp7b1	F: 5'-TGAGGTTCTGAGGCTGTGC-3'<br>R: 5'-TGGAGGAAAGAGGGCTACAA-3'
Fxr	F: 5'-CCCCTGCTTGATGTGCTAC-3'<br>R: 5'-CGTGGTGATGGTTGAATGTC-3'
Shp	F: 5'-AAGGGCACGATCCTCTTCAA-3'<br>R: 5'-CTGTTGCAGGTGTGCGATGT-3'
Fgfr4	F: 5'-GCATCTTTCAGGGGACACCA-3'<br>R: 5'-TTGTACCAGTGACGACCACG-3'
Ucp1	F: 5'-GCTACACGGGGACCTACAATG-3'<br>R: 5'-CGTCATCTGCCAGTATTTTGTT- 3'
Pgc1α	F: 5'-AGCCGTGACCACTGACAACGAG -3'<br>R: 5'- GCTGCATGGTTCTGAGTGCTAAG - 3'
Elovl3	F: 5'-TTCTCACGCGGGTTAAAAATGG-3'<br>R: 5'-GAGCAACAGATAGACGACCAC-3'
Elovl6	F: 5'-AAAGCACCCGAACTAGGTGA-3'<br>R: 5'-AGGAGCACAGTGATGTGGTG-3'

Abbreviations: glyceraldehyde-3-phosphate dehydrogenase (*Gapdh*); Takeda G protein-coupled receptor 5 (*Tgr5*); cytochrome P450-27A1 (*Cyp27a1*); cytochrome P-450 cholesterol 7α-hydroxylase (*Cyp7a1*); sterol-12α-hydroxylase (*Cyp8b1*); oxysterol 7α-hydroxylase (*Cyp7b1*); farnesoid X receptor (*Fxr*); small heterodimer partner (*Shp*); fibroblast growth factor receptor 4 (*Fgfr4*); uncoupling protein 1 (*Ucp1*); peroxisome proliferator-activated receptor-γ coactivator-1α (*Pgc1α*); elongation of very long chain fatty acids 3 (*Elovl3*); elongation of very long chain fatty acids 6 (*Elovl6*).

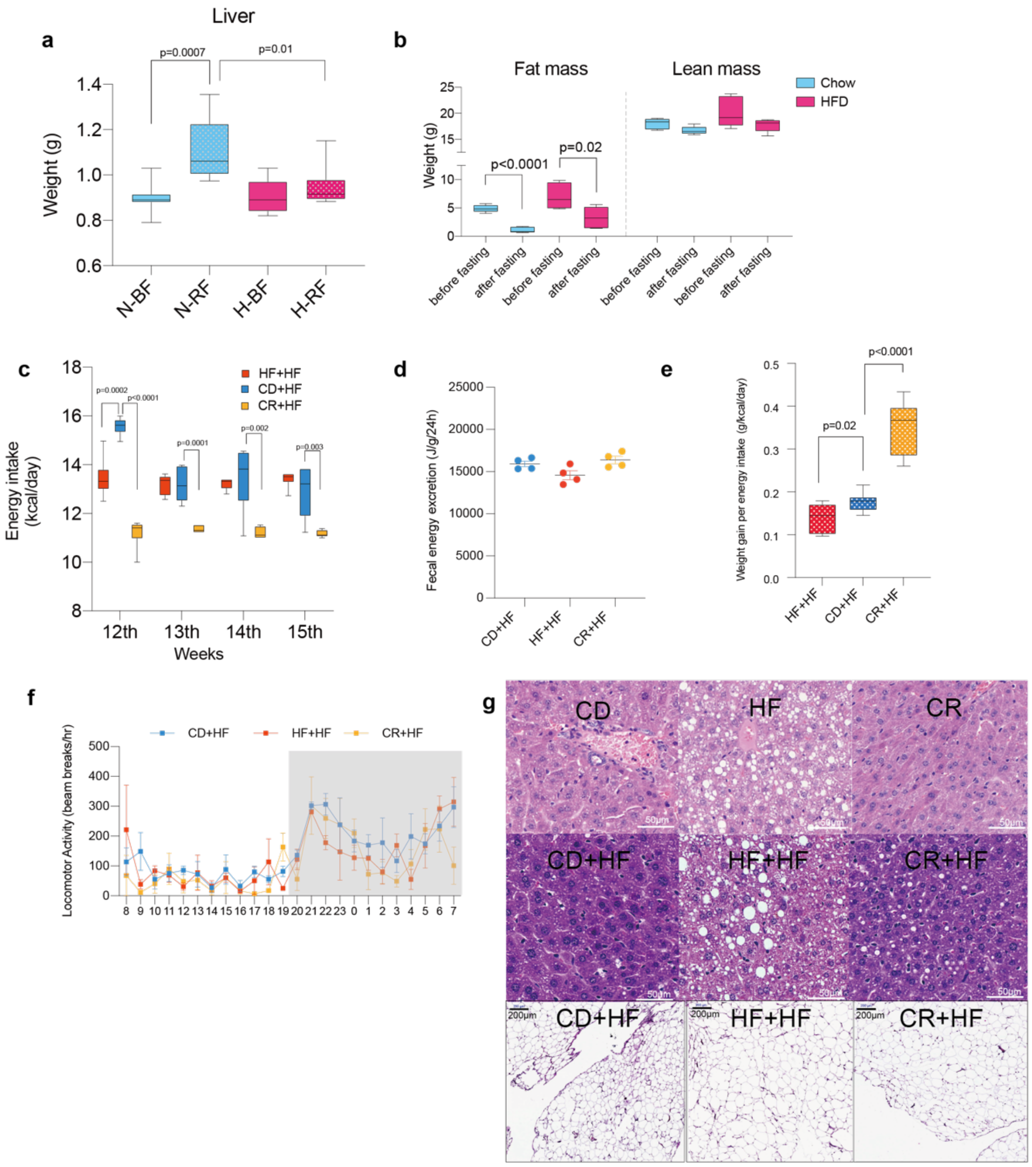

Supplementary Fig. 1. a The liver weights of the four groups in the fasting experiments. **b** The body composition in the fasting experiments (n=4 per group). **c** The energy intake per day per mouse in the diets changing experiments (n=6 per group). **d** The fecal energy excretion per day in the diets changing experiments (n=4 per group). **e** The energy intake per day per weight gain in the diets changing experiments. **f** The locomotor activity in three groups (n=4 per group). Differences were assessed by the two-way analysis of variance (ANOVA), p=0.39. **g** Representative images of H&E staining of the liver and WAT section, three times in each experiment were repeated independently with similar results. n=8 per group. All p values in figures were calculated by the two-tailed unpaired T-test and ANOVA in the GraphPad software. Data are expressed as means±SEM in the bar plots. All box and whiskers plots showed the box (from the 25th to 75th percentiles), the median value (in the transverse line), and the whiskers (go down to the smallest value and up to the largest). Source data are provided as a Source Data file.

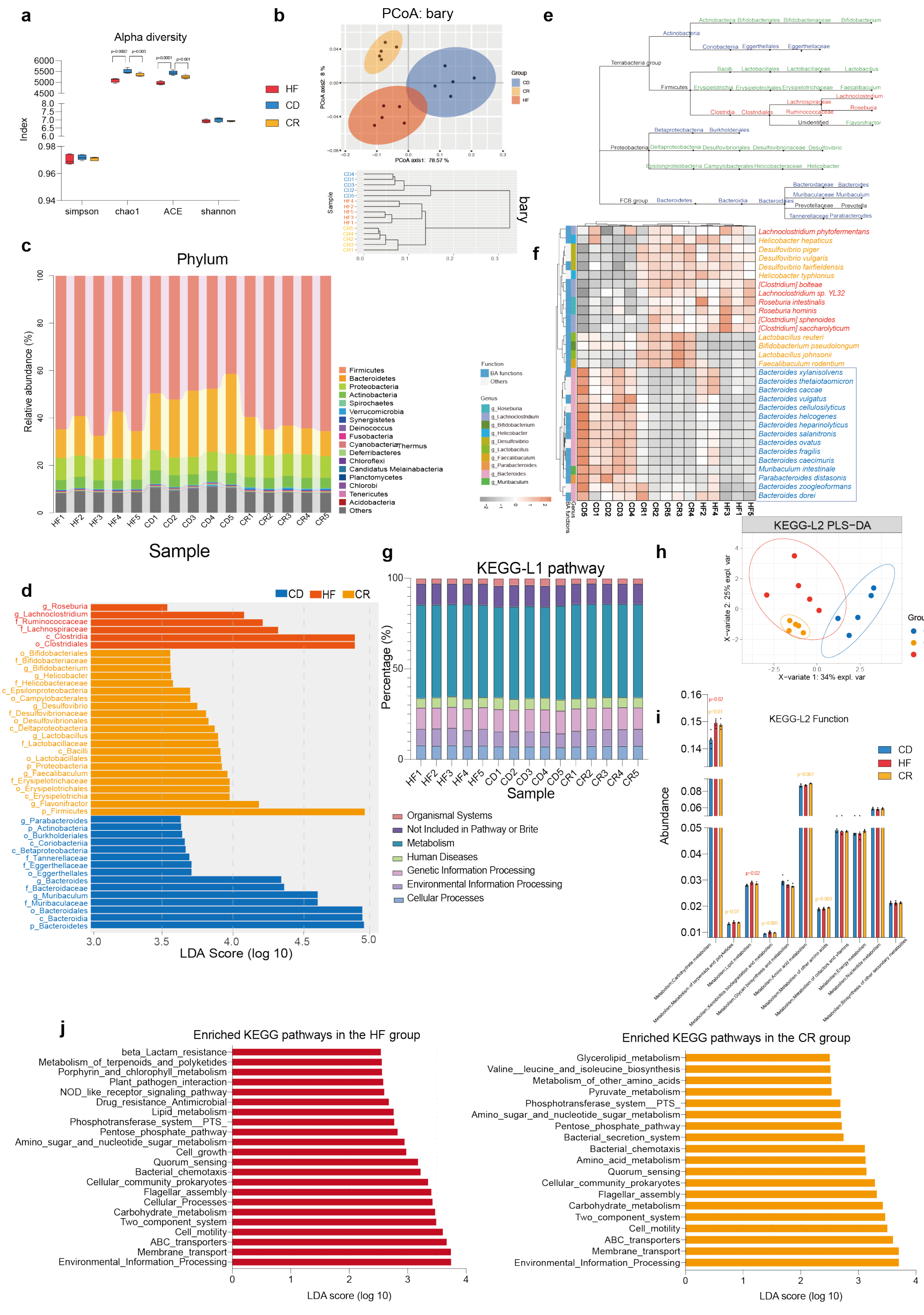

Supplementary Fig. 2. a The alpha diversity index in HF, CD, and CR groups. **b** Principal coordinate analysis (PCoA) plot and hierarchical clustering based on the Bray–Curtis similarity. **c** Relative abundance of each phylum in each sample. **d** Linear discriminant analysis (LDA) effect size method was performed to compare taxa (levels from phylum to genus) among three groups. The bar plot lists the significantly differential taxa (LDA score (log 10) > 3.5). Enriched taxa in HF (red), CR (orange) and Chow (blue) diets. **e** Phylogenetic tree of enriched taxa in LDA analysis. The taxa in red indicate increased abundance in HF group, blue indicate increased abundance in CD

group, and green indicate increased abundance in CR group. **f** Heatmap of relative abundance of representative species, which belong to the enriched genus in HF (red), CR (yellow) and CD (blue) groups. BA functions include the function of bile salt hydrolase, 7-alpha-hydroxysteroid dehydrogenase or other bile acids related functions. **g** KEGG L1 pathways relative abundance in each sample. **h** The PLS-DA plot by the KEGG L2 pathways of the "Metabolism" pathway. **i** The KEGG L2 pathways abundance of the Metabolism function among the three groups. Differences between the CD group and the CR group are marked in yellow. Differences between the CD group and the HF group are marked in red. **j** The enriched KEGG pathway in HF and CR groups. n=5 per group. All p values in figures were calculated by the two-tailed unpaired T-test in the GraphPad software. Data are expressed as means±SEM in the bar plots. Source data are provided as a Source Data file. Abbreviations: Principal coordinate analysis (PCoA); bile acid (BA); linear discriminant analysis (LDA); partial least-squares discriminant analysis (PLS-DA).

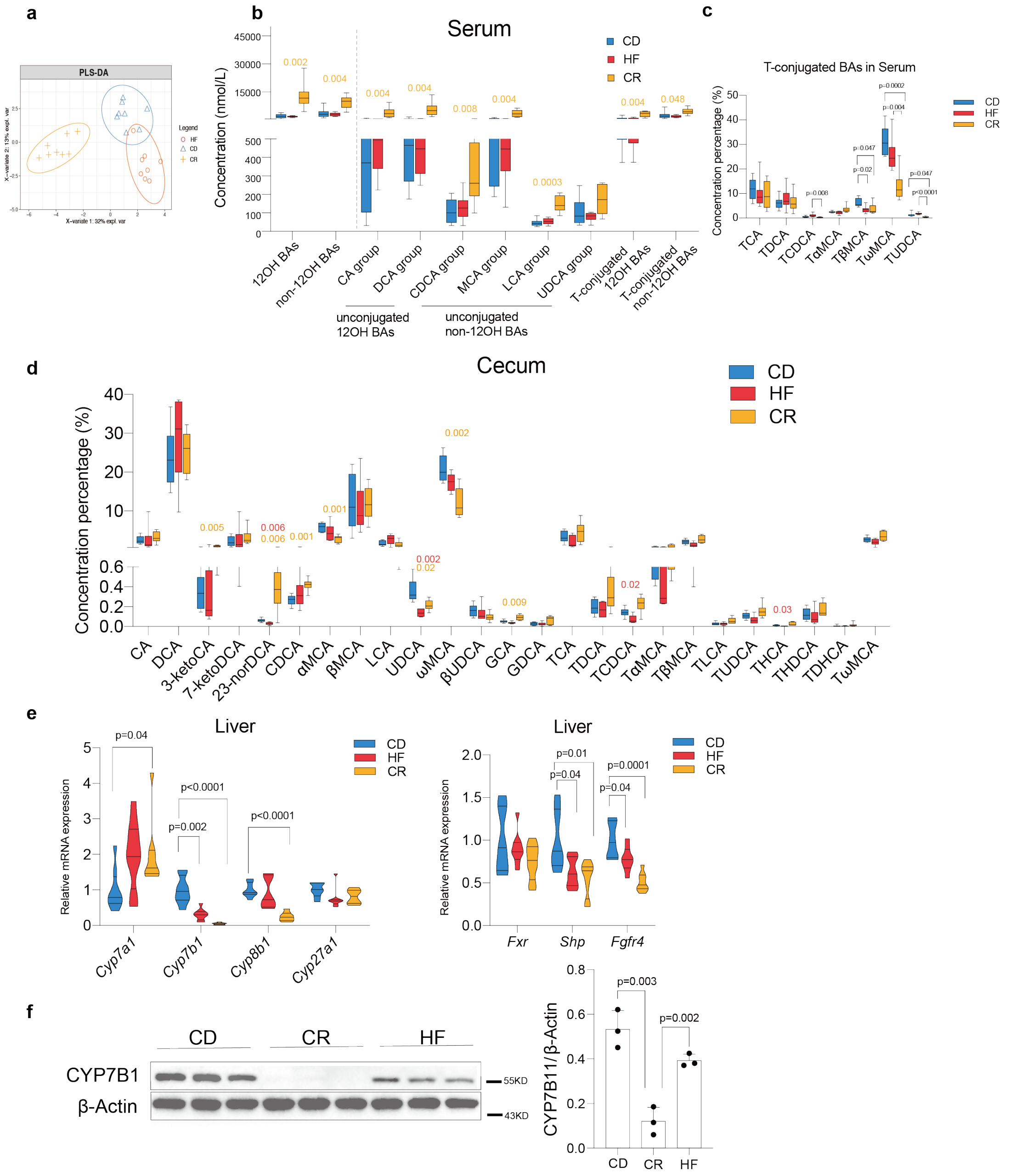

Supplementary Fig. 3. a The PLS-DA plots of the serum BAs in HF, CD, and CR groups. **b** The concertation level of BAs in serum. p values marked in yellow represent the differences between the CD group and the CR group. **c** The T-conjugated BAs profiles in the serum among three groups. **d** The BAs composition profile in the cecal contents. p values marked in yellow represent the differences between the CD group and the CR group. p values marked in red represent the differences between the CD group and the HF group. **e** The major BAs synthesis-related enzymes and *Fxr* signaling pathway mRNA expression in liver. **f** The expression of CYP7B1 in western blots, n=3. n=8 per group in the experiment. All p values in figures were calculated by the two-tailed unpaired T-test in the GraphPad software. p-values were adjusted by the FDR's method. Data are expressed as means±SEM in the bar plots. All box and whiskers plots showed the box (from the 25th to 75th percentiles), the median value (in the transverse line), and the whiskers (go down to the smallest value and up to the largest). Source data are provided as a Source Data file.

Abbreviations: partial least-squares discriminant analysis (PLS-DA); 12α-hydroxylated bile acids (12OH BAs); non-12α-hydroxylated bile acids (non-12OH BAs); cholic acid (CA); deoxycholic acid (DCA); chenodeoxycholic acid (CDCA); muricholic acid (MCA); lithocholic acid (LCA); ursodeoxycholic acid (UDCA); bile acids (BAs); taurocholic acid (TCA); taurodeoxycholic acid (TDCA); taurochenodeoxycholic acid (TCDCA); tauro α-muricholic acid (TαMCA); tauro β-muricholic acid (TβMCA); tauro ω-muricholic acid (TωMCA); tauroursodeoxycholic acid (TUDCA); 3-ketocholic acid (3-ketoCA); 7-ketodexycholic acid (7-ketoDCA); 23-nordeoxycholic acid (23-norDCA); α-muricholic acid (αMCA); β-muricholic acid (βMCA); ω-muricholic acid (ωMCA); 3β-ursodeoxycholic acid (βUDCA); glycocholic acid (GCA); glycodehydrocholic acid (GDCA); taurolithocholic acid (TLCA); taurohyocholic acid (THCA); taurohyodeoxycholic acid (THDCA); taurodehydrocholic acid (TDHCA); cytochrome P-450 cholesterol 7α-hydroxylase (*Cyp7a1*); oxysterol 7α-hydroxylase (*Cyp7b1*); sterol-12α-hydroxylase (*Cyp8b1*); cytochrome P450-27A1 (*Cyp27a1*); farnesoid X receptor (*Fxr*); small heterodimer partner (*Shp*); fibroblast growth factor receptor 4 (*Fgfr4*).

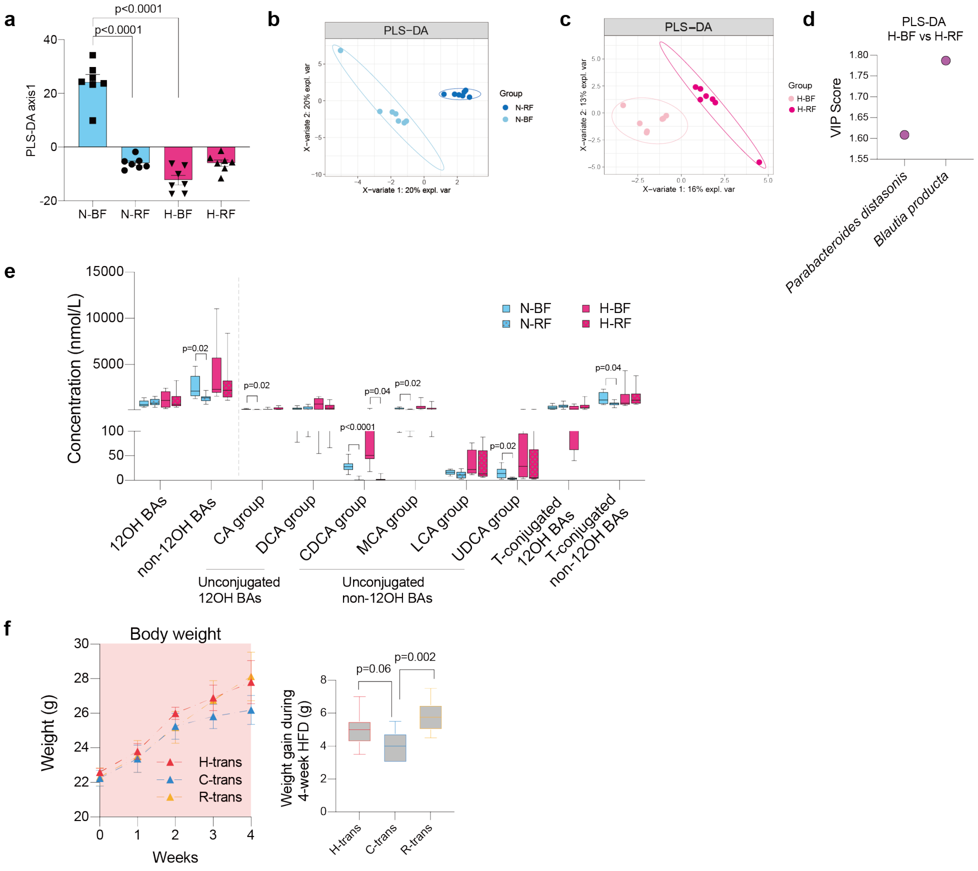

Supplementary Fig. 4. a The axis1 level of PLS-DA plot of cecal microbial composition in four groups. **b** The PLS-DA plot on species level in the normal chow diet groups. **c** The PLS-DA plot on species level in the HFD groups. **d** The species rank of VIP scores of the PLS-DA analysis in HFD groups. n=9 per group. **e** The concertation level of BAs in serum. **f** Receiver mice weight gain during HFD. All p values in figures were calculated by the two-tailed unpaired T-test in the GraphPad software. p-values were adjusted by the FDR's method. Data are expressed as means±SEM in the bar plots. All box and whiskers plots showed the box (from the 25th to 75th percentiles), the median value (in the transverse line), and the whiskers (go down to the smallest value and up to the largest). Source data are provided as a Source Data file. Abbreviations: partial least-squares discriminant analysis (PLS-DA); 12α-hydroxylated bile acids (12OH BAs); non-12α-hydroxylated bile acids (non-12OH BAs); cholic acid (CA); deoxycholic acid (DCA); chenodeoxycholic acid (CDCA); muricholic acid (MCA); lithocholic acid (LCA); ursodeoxycholic acid (UDCA); bile acids (BAs).

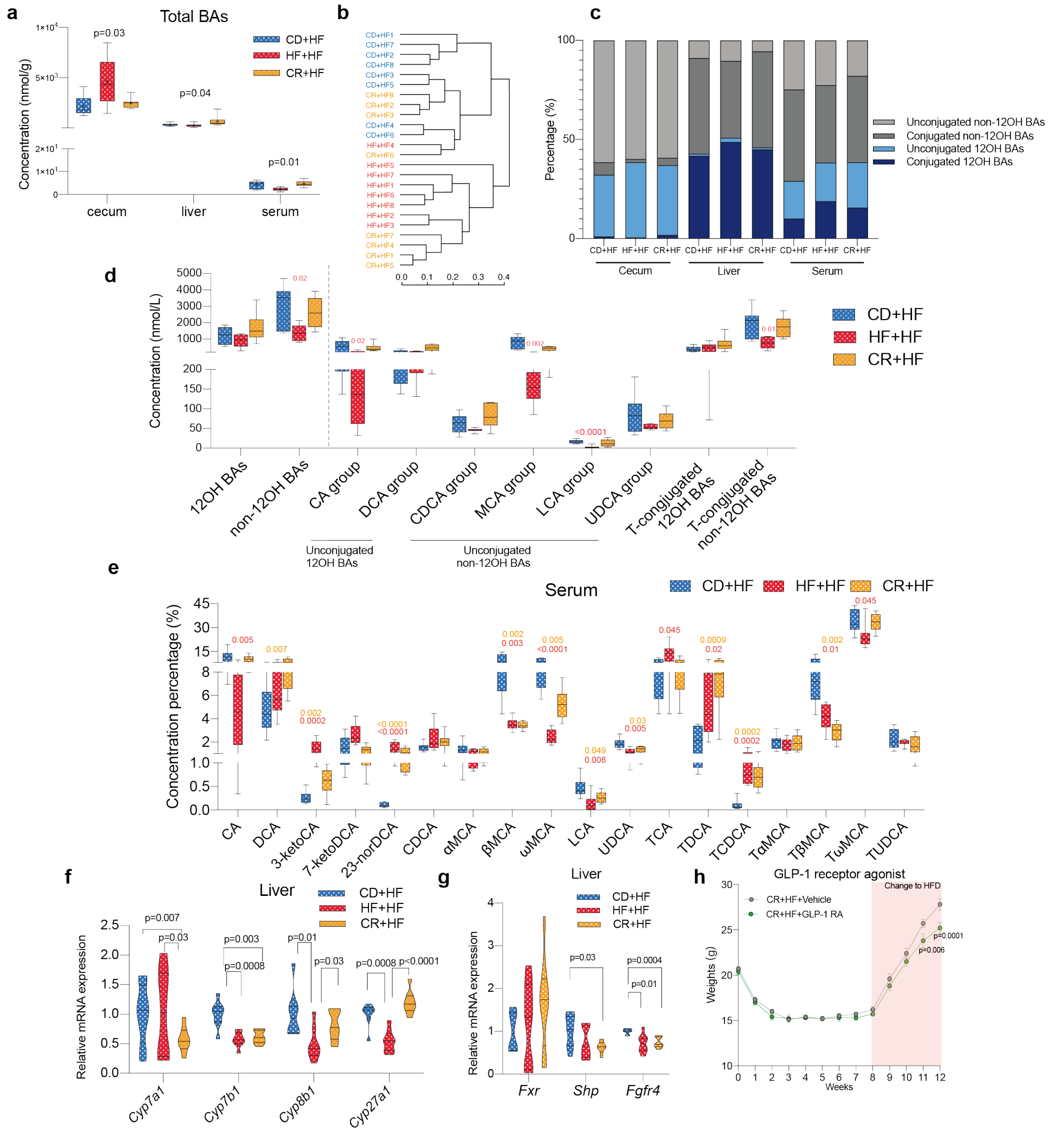

Supplementary Fig. 5. a The total BAs concentration of the contents of cecum, liver, serum in CD+HF, HF+HF, CR+HF groups. **b** Hierarchical clustering based on the Canberra similarity among the three groups. **c** The bar plots show the mean percentage of (non-)12OH and (un)conjugated BAs in cecum, liver and serum among three groups. **d** The BAs composition profile in the serum. p values marked in red represent the differences between CD+HF group and the HF+HF group. **e** The concertation level of BAs in serum. p values marked in yellow represent the differences between CD+HF group and the CR+HF group. p values marked in red represent the differences between CD+HF group and the HF+HF group. **f** The mRNA expression of 4 major BAs synthesis-related liver enzymes. **g** *Fxr* signaling pathway mRNA expression in liver. n=8 per group. **h** Body weights in the GLP-1 receptor agonist experiments. All p values in figures were calculated by the two-tailed unpaired T-test in the GraphPad software. p-values were adjusted by the FDR's method. Data are expressed as means±SEM in the bar plots. All box and whiskers plots showed the box (from the 25th to 75th percentiles), the median value (in the transverse line), and the whiskers (go down to the smallest value and up to the largest). Source data are provided as a Source Data file. Abbreviations: bile acids (BAs); 12α-hydroxylated bile acids (12OH BAs); non-12α-hydroxylated

bile acids (non-12OH BAs); cholic acid (CA); deoxycholic acid (DCA); chenodeoxycholic acid (CDCA); muricholic acid (MCA); lithocholic acid (LCA); ursodeoxycholic acid (UDCA); taurocholic acid (TCA); taurodeoxycholic acid (TDCA); taurochenodeoxycholic acid (TCDCA); tauro α-muricholic acid (TαMCA); tauro β-muricholic acid (TβMCA); tauro ω-muricholic acid (TωMCA); tauroursodeoxycholic acid (TUDCA); 3-ketocholic acid (3-ketoCA); 7-ketodexycholic acid (7-ketoDCA); 23-nordeoxycholic acid (23-norDCA); α-muricholic acid (αMCA); β-muricholic acid (βMCA); ω-muricholic acid (ωMCA); cytochrome P-450 cholesterol 7α-hydroxylase (*Cyp7a1*); oxysterol 7α-hydroxylase (*Cyp7b1*); sterol-12α-hydroxylase (*Cyp8b1*); cytochrome P450-27A1 (*Cyp27a1*); farnesoid X receptor (*Fxr*); small heterodimer partner (*Shp*); fibroblast growth factor receptor 4 (*Fgfr4*).

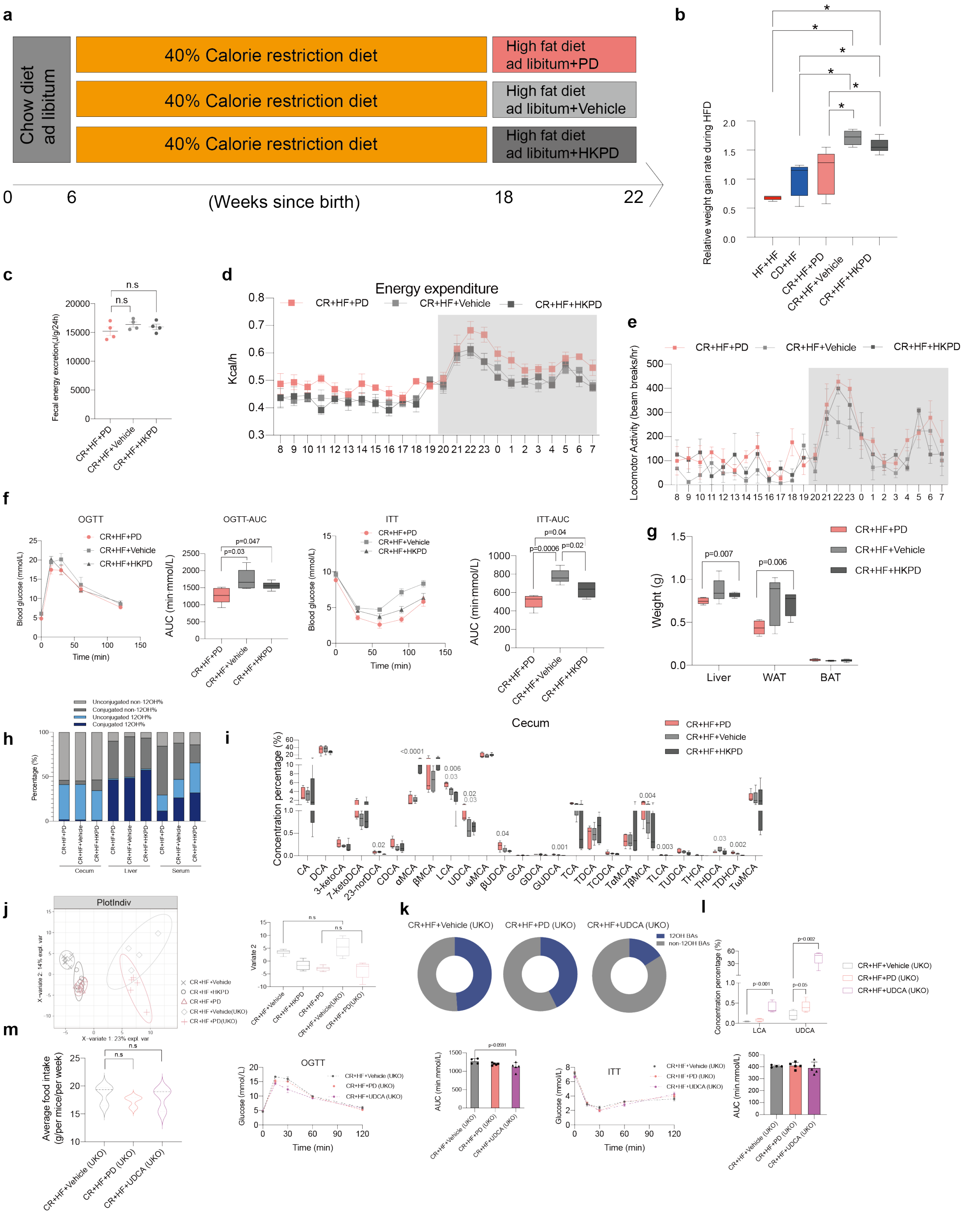

Supplementary Fig. 6. a The experimental workflow of *Parabacteroides distasonis* intervention. **b** The relative weight gain during the period of changing to HFD. **c** The fecal energy excretion per day in the experiment (n=4 per group). **d** Raw energy expenditures and raw average energy expenditures during the period of light and dark. **e** The locomotor activity in three groups. n=4 per group. Differences were assessed by the two-way analysis of variance (ANOVA), p=0.19. **f** Blood glucose and AUC in the OGTT. Blood glucose and AUC in the ITT. **g** The tissues weight. **h** The bar plots show the mean percentage of (non-)12OH and (un)conjugated BAs in cecum, liver and serum among three groups. **i** The BAs composition profile in the cecal contents. p values marked in light gray represent the differences between CR+HF+PD group and the CR+HF+Vehicle group. p values marked in dark gray represent the differences between CR+HF+PD group and the CR+HF+HKPD

group. **j** The PLS-DA plot (left) and the variate 2 (right, the variate of y-axis in the PLS-DA plot) of microbial metabolites profiling in WT and UKO mice serum, n=8 in the CR+HF+PD and CR+HF+Vehicle groups. n=5 in the CR+HF+HKPD and CR+HF+PD (UKO) groups. n=4 in the CR+HF+ Vehicle (UKO) group. **k** The percentage of 12OH BAs /non-12OH BAs, **l** LCA, and UDCA (right) in serum. **m** The average food intake and blood glucose in OGTT and ITT and AUCs in UKO mice. n=5 per group. All p values in figures were calculated by the two-tailed unpaired T-test in the GraphPad software. p-values were adjusted by the FDR's method. Data are expressed as means±SEM in the bar plots. All box and whiskers plots showed the box (from the 25th to 75th percentiles), the median value (in the transverse line), and the whiskers (go down to the smallest value and up to the largest). Source data are provided as a Source Data file. Abbreviations: oral glucose tolerance test (OGTT); insulin tolerance tests (ITT); area under the curve (AUC); white adipose tissue (WAT); brown adipose tissue (BAT); cholic acid (CA); deoxycholic acid (DCA); 3-ketocholic acid (3-ketoCA); 7-ketodexycholic acid (7-ketoDCA); 23-nordeoxycholic acid (23-norDCA); chenodeoxycholic acid (CDCA); α-muricholic acid (αMCA); β-muricholic acid (βMCA); lithocholic acid (LCA); ursodeoxycholic acid (UDCA); ω-muricholic acid (ωMCA); 3β-ursodeoxycholic acid (βUDCA); glycocholic acid (GCA); glycodehydrocholic acid (GDCA); taurocholic acid (TCA); taurodeoxycholic acid (TDCA); taurochenodeoxycholic acid (TCDCA); tauro α-muricholic acid (TαMCA); tauro β-muricholic acid (TβMCA); taurolithocholic acid (TLCA); tauroursodeoxycholic acid (TUDCA); taurohyocholic acid (THCA); taurohyodeoxycholic acid (THDCA); taurodehydrocholic acid (TDHCA); tauro ω-muricholic acid (TωMCA); 12α-hydroxylated bile acids (12OH BAs); non-12α-hydroxylated bile acids (non-12OH BAs).

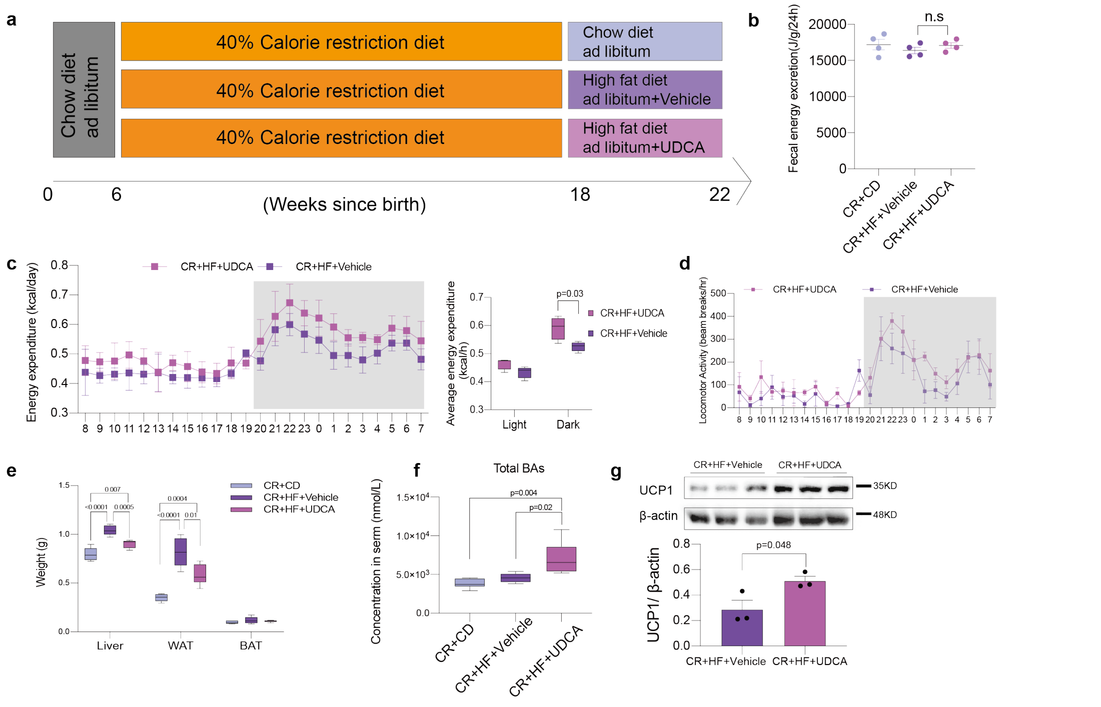

Supplementary Fig. 7. a The experimental workflow of UDCA intervention. **b** The fecal energy excretion per day in the experiment (n=4 per group). **c** Raw energy expenditures and raw average energy expenditures during the period of light and dark. **d** The locomotor activity in three groups. n=4 per group. Differences were assessed by the two-way analysis of variance (ANOVA), p=0.17. **e** Tissues weights. **f** The total BAs concentration of the serum, n=6 per group. **g** The expression of UCP1 in western blots, n=3. All p values in figures were calculated by the two-tailed unpaired T-test in the GraphPad software. p-values were adjusted by the FDR's method. Data are expressed as means±SEM in the bar plots. All box and whiskers plots showed the box (from the 25th to 75th percentiles), the median value (in the transverse line), and the whiskers (go down to the smallest value and up to the largest). Source data are provided as a Source Data file. Abbreviations: ursodeoxycholic acid (UDCA); white adipose tissue (WAT); brown adipose tissue (BAT); bile acid (BA); uncoupling protein 1 (UCP1).
