# Supplementary material for: Gut microbiota-bile acid crosstalk contributes to the rebound weight gain after calorie restriction in mice
Source: Nat Commun. 2022 Apr 19;13:2060. doi: 10.1038/s41467-022-29589-7 (PMC9018700; doi:10.1038/s41467-022-29589-7)
Supplement: Supplementary file 3 — Reporting Summary [file 41467_2022_29589_MOESM3_ESM.pdf]

nature portfolio

Corresponding author(s): Wei Jia

Last updated by author(s): Mar 9, 2022

Reporting Summary

Nature Portfolio wishes to improve the reproducibility of the work that we publish. This form provides structure for consistency and transparency in reporting. For further information on Nature Portfolio policies, see our Editorial Policies and the Editorial Policy Checklist.

Statistics

For all statistical analyses, confirm that the following items are present in the figure legend, table legend, main text, or Methods section.

n/a	Confirmed	
☐	☒	The exact sample size (n) for each experimental group/condition, given as a discrete number and unit of measurement
☐	☒	A statement on whether measurements were taken from distinct samples or whether the same sample was measured repeatedly
☐	☒	The statistical test(s) used AND whether they are one- or two-sided<br>*Only common tests should be described solely by name; describe more complex techniques in the Methods section.*
☐	☒	A description of all covariates tested
☐	☒	A description of any assumptions or corrections, such as tests of normality and adjustment for multiple comparisons
☐	☒	A full description of the statistical parameters including central tendency (e.g. means) or other basic estimates (e.g. regression coefficient) AND variation (e.g. standard deviation) or associated estimates of uncertainty (e.g. confidence intervals)
☐	☒	For null hypothesis testing, the test statistic (e.g. F, t, r) with confidence intervals, effect sizes, degrees of freedom and P value noted<br>*Give P values as exact values whenever suitable.*
☒	☐	For Bayesian analysis, information on the choice of priors and Markov chain Monte Carlo settings
☒	☐	For hierarchical and complex designs, identification of the appropriate level for tests and full reporting of outcomes
☐	☒	Estimates of effect sizes (e.g. Cohen's d, Pearson's r), indicating how they were calculated

Our web collection on statistics for biologists contains articles on many of the points above.

Software and code

Policy information about availability of computer code

Data collection	The softwares of MassLynx v4.1 and TargetLynx v4.1 (Waters, Milford, MA), Agilent MassHunter (SCN 712, Agilent Technologies, USA) were used for data collection.
Data analysis	R (R version 3.6.3), MEGAN (MEtaGenome Analyzer) software, QIIME (http://qiime.org/scripts/pick_otus.html), GraphPad Prism (8.4.0, Graphpad, USA), ImageJ (version 1.53), IP4M 2.0 (http://ip4m.cn) were used for statistical analyses.

For manuscripts utilizing custom algorithms or software that are central to the research but not yet described in published literature, software must be made available to editors and reviewers. We strongly encourage code deposition in a community repository (e.g. GitHub). See the Nature Portfolio guidelines for submitting code & software for further information.

Data

Policy information about availability of data

All manuscripts must include a data availability statement. This statement should provide the following information, where applicable:

- Accession codes, unique identifiers, or web links for publicly available datasets
- A description of any restrictions on data availability
- For clinical datasets or third party data, please ensure that the statement adheres to our policy

Source data are provided with this paper. The 16S rRNA gene sequences and metagenomic sequences were provided and available at National Center for Biotechnology Information Sequence Read Archive (SRP) database with accession code PRJNA813171 and PRJNA813023. The metabolomics data were deposited and available at MetaboLights repository with accession code MTBLS4431.

Field-specific reporting

Please select the one below that is the best fit for your research. If you are not sure, read the appropriate sections before making your selection.

☒ Life sciences ☐ Behavioural & social sciences ☐ Ecological, evolutionary & environmental sciences

For a reference copy of the document with all sections, see nature.com/documents/nr-reporting-summary-flat.pdf

Life sciences study design

All studies must disclose on these points even when the disclosure is negative.

Sample size	The sample sizes were predetermined by IP4M 2.0. The power was set as 0.85 (significant level 0.05, two tails) and the effect size was set as 0.5.
Data exclusions	No data were excluded from the analyses.
Replication	We have verify the reproducibility of experimental findings. These results were verified by animal (n=5-9/group) studies involved in the manuscripts.
Randomization	The mice were randomly divided and allocated into experimental groups.
Blinding	The investigators were blinded to the group allocation during data collection and pretreatment. The instrument running order was randomized. Investigators knew the group allocation after raw data pretreatment for statistical analysis.

Reporting for specific materials, systems and methods

We require information from authors about some types of materials, experimental systems and methods used in many studies. Here, indicate whether each material, system or method listed is relevant to your study. If you are not sure if a list item applies to your research, read the appropriate section before selecting a response.

Materials & experimental systems

n/a	Involved in the study	
☐	☒	Antibodies
☒	☐	Eukaryotic cell lines
☒	☐	Palaeontology and archaeology
☐	☒	Animals and other organisms
☒	☐	Human research participants
☒	☐	Clinical data
☒	☐	Dual use research of concern

Methods

n/a	Involved in the study	
☒	☐	ChIP-seq
☒	☐	Flow cytometry
☒	☐	MRI-based neuroimaging

Antibodies

Antibodies used	UCP1(1:1000, Cat: #14670, Cell Signaling Technology, Beverly, MA), CYP7B1 (1:1000, Cat: ab138497, Abcam, Cambridge, United Kingdom), β-Actin (1:1000, Cat: #4970, Cell Signaling Technology, Beverly, MA), secondary antibodies (Anti-rabbit IgG, Cat: #7074, Cell Signaling Technology, Beverly, MA).
Validation	The antibodies used in our study are for western-blot (WB) staining. The quality of the antibodies are all guaranteed by the manufacturers, and we further validated the antibody in varied assays for WB detection.

Animals and other organisms

Policy information about studies involving animals; ARRIVE guidelines recommended for reporting animal research

Laboratory animals	C57BL/6J male mice at the age of 6 weeks and 6-8 weeks C57BL/6J female mice were involved in this study.
Wild animals	No
Field-collected samples	No
Ethics oversight	The animal studies were approved by the Institutional Animal Care and Use Committee of Shanghai Jiao Tong University Affiliated Sixth People's Hospital, the animal welfare ethics acceptance number is DWLL2020-0571.

Note that full information on the approval of the study protocol must also be provided in the manuscript.
